# *KRAS* mutation detection by liquid biopsy for pancreatic ductal adenocarcinoma

**DOI:** 10.1186/s13045-025-01696-0

**Published:** 2025-04-17

**Authors:** Mahmoud Yousef, Abdelrahman Yousef, Mark W. Hurd, Ashwathy Pillai, Saikat Chowdhury, Rebecca Snyder, Mark Knafl, Ryan L. Lewis, Paul M. Roy, Mohammad Fanaeian, Sali Albarouki, Luca F. Castelnovo, Jennifer Peterson, Brandon G. Smaglo, Robert A. Wolff, Shubham Pant, Jason Willis, Ryan Huey, Michael Overman, Ching-Wei Tzeng, Michael P. Kim, Naruhiko Ikoma, Jess E. Maxwell, Matthew H. G. Katz, Huamin Wang, Anirban Maitra, Eugene Koay, Ethan B. Ludmir, Anthony Chen, Camila Lopez, Haoqiang Ying, John Paul Shen, Dan Zhao

**Affiliations:** 1 Department of Gastrointestinal Medical Oncology, The University of Texas MD Anderson Cancer Center, Houston, TX USA; 2 Sheikh Ahmed Center for Pancreatic Cancer Research, The University of Texas MD Anderson Cancer Center, Houston, TX USA; 3 Department of Hospital Medicine, The University of Texas MD Anderson Cancer Center, Houston, TX USA; 4 Department of Surgical Oncology, The University of Texas MD Anderson Cancer Center, Houston, TX USA; 5 Department of Genomic Medicine, The University of Texas MD Anderson Cancer Center, Houston, TX USA; 6 Department of Enterprise Data Engineering and Analytics, The University of Texas MD Anderson Cancer Center, Houston, TX USA; 7 Department of Gastroenterology and Hepatology, Baylor College of Medicine, Houston, TX USA; 8 Department of Anatomical Pathology, The University of Texas MD Anderson Cancer Center, Houston, TX USA; 9 Department of Gastrointestinal Radiation Oncology, The University of Texas MD Anderson Cancer Center, Houston, TX USA; 10 Department of Biostatistics, The University of Texas MD Anderson Cancer Center, Houston, TX USA; 11 Department of Molecular and Cellular Oncology, Division of Basic Science Research, The University of Texas MD Anderson Cancer Center, Houston, TX USA

**Keywords:** Pancreatic cancer, Liquid biopsy, KRAS, PDAC, Molecular profiling, Mutation, OS

## Abstract

The clinical utility of liquid biopsy (LB) for pancreatic ductal adenocarcinoma (PDAC) remain understudied. Our single-institution cohort of 311 PDAC patients with non-tumor tissues informed LB found 81.2% positivity (*N* = 186) in metastatic cases and in 52.4% (*N* = 43) of localized disease. *KRAS* mutations were detected in 64.6% (*N* = 148) of metastatic cases and 16% (*N* = 13) for localized disease. Positive LB, especially *KRAS* mutation detection, is associated with worse overall survival (OS) in metastatic PDAC (median 14.5 vs. 31.3 months, HR = 2.7, 95%CI = 1.7–4.3, *P* < 0.0001). The positive concordance rates of *KRAS* and *TP53* mutations were 63% and 68% in metastatic disease but only 7% (*KRAS*) and 33% (*TP53*) in localized disease, respectively. Among the 41 patients who underwent serial liquid biopsy testing, 25% tested positive after an initial negative result. LB detects therapeutically targetable mutations in 58.5% of PDAC patients and is associated with OS.

**Supplementary Information:**

The online version contains supplementary material available at 10.1186/s13045-025-01696-0.

## To the editor

*KRAS* is mutated in approximately 90% of pancreatic ductal adenocarcinoma (PDAC) including 35% *KRAS*^G12D^, 30% *KRAS*^G12V^, 15% *KRAS*^G12R^, and 1-2% *KRAS*^G12C^ [1, 2]. KRAS^G12C^ inhibitors showed efficacy in PDAC and many KRAS inhibitors are in clinical development [3–8]. We previously reported the *KRAS* mutation by tissue testing with PDAC outcome which is associated with worse overall survival (OS) [9]. The utility of liquid biopsy (LB) is promising in PDAC [10, 11]. CtDNA positive rate was 29.48% by tumor-informed whole exome sequencing (WES) in post-surgical PDAC patients on surveillance [12]. There are few real-world data on the non-tumor tissue informed liquid biopsy testing.

## Results

**Fig. 1:**
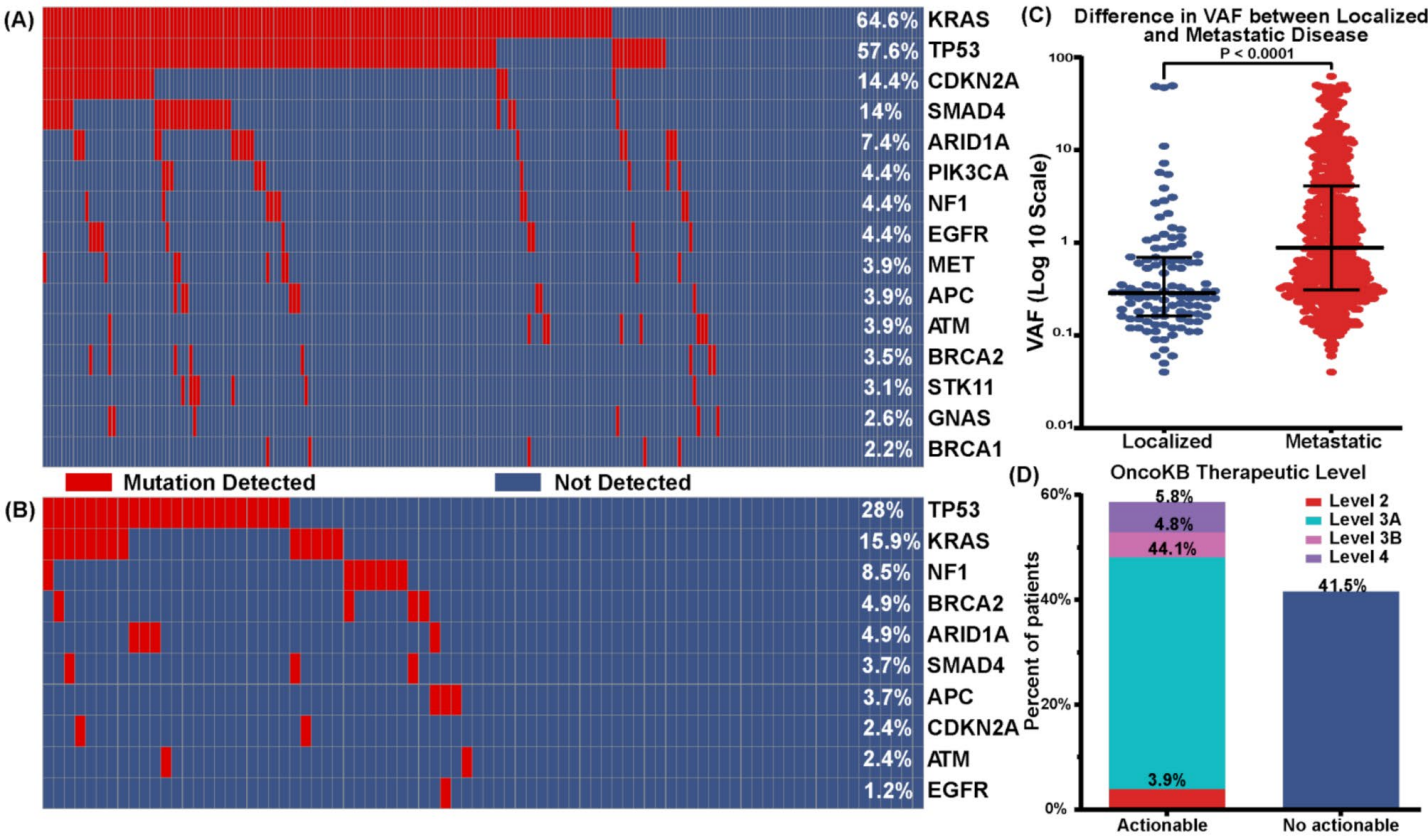
Mutations detected in LB and OS. **A**- Oncoplot for mutations detected in metastatic disease at LB. **B**- Oncoplot for mutations detected in localized disease at LB. **C**- Difference in median VAF of mutations detected in LB between localized disease and metastatic disease. **D**-Rates of actionable mutations detected in LB by OncoKB therapeutic levels

We analyzed 311 PDAC patients underwent in-house non tumor informed ctDNA testing from 2018 to 2023 at MD Anderson cancer center. 73% (*N* = 229) had metastatic disease (Supplemental Methods, Table S1). The median follow-up was 34.9 months with median OS 22.5 months (95% CI = 19.2–25.8). The median age at diagnosis was 64.9 years old. LB was positive in 81.2% (*N* = 186) of metastatic cases 52.4% (*N* = 43) of localized disease. *KRAS* mutations were detected in 64.6% (*N* = 148) metastatic disease, followed by *TP53* (57.6%, *N* = 132, Fig. 1-A). However, for localized disease, the most detected mutation was *TP53* (28%, *N* = 23), followed by *KRAS* (16%, *N* = 13) (Fig. 1-B). Median VAF in localized disease was significantly lower than metastatic disease, medians (interquartile range) = 0.29 (0.53) vs. 0.88 (3.78) respectively, *P* < 0.001, Fig. 1-C). LB detected actionable mutations in 58.5% (*N* = 182) of all patients tested according to the OncoKB therapeutic level of evidence classification, with 3.9% (*N* = 12) at level 2, 44.1% (*N* = 137) at level 3 A, 4.8% (*N* = 15) at level 3 B, and 5.8% (*N* = 18) at level 4 (Fig. 1-D). The positive concordance rate for the subset of patients underwent tissue biopsy testing (*n* = 116), was 63% (*n* = 50/80) for *KRAS* mutation, 68% (*n* = 43/63) for *TP53* mutation, 26% (*n* = 5/19) for *SMAD4*, and 80% (*n* = 8/10) for *CDKN2A* in metastatic disease. Localized disease had lower positive concordance rate, with 7% (*n* = 2/27) for *KRAS* and 33% (*n* = 7/21) for *TP53* (Table S2-3).

**Fig. 2:**
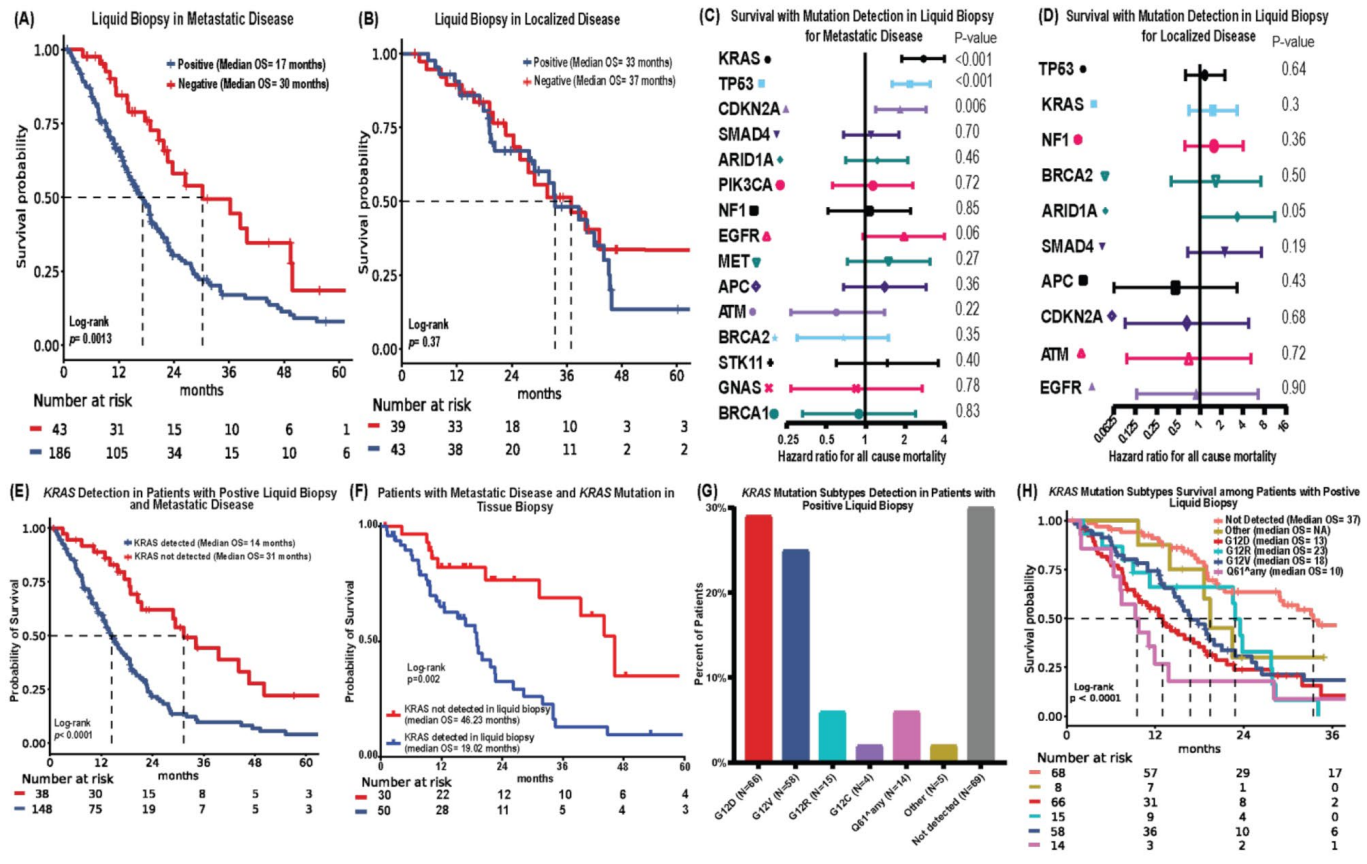
Outcomes with positivity of LB and with *KRAS* mutations detection. **A**- OS with positive LB in metastatic disease. **B**- OS with positive LB in localized disease. **C**- Hazard ratios (HRs) of OS with mutation detection by LB in metastatic disease. **D**- HRs of OS with mutation detection in LB in localized disease. **E**- OS of patients with positive *KRAS* mutation vs. other mutations in LB for metastatic disease. **F**- OS of metastatic disease patients with positive *KRAS* mutation detected by tissue NGS stratified by *KRAS* mutation detection in LB. **G**- Frequencies of detected *KRAS* mutation subtypes. **H**- OS of patients with *KRAS* mutation detected in LB by *KRAS* mutation subtypes

Positive LB was associated with worse OS (HR = 2.1, 95%CI = 1.3–3.3, *P* = 0.0015) in metastatic disease (Fig. 2A). The OS difference was not significant (HR = 1.3, 95%CI = 0.72–2.5, *P* = 0.36; Fig. 2B) in localized disease. Univariate COX regression analyses for OS in metastatic cases showed that mutations in *KRAS* (HR = 2.8, 95%CI = 1.9-4, *P* < 0.001), *TP53* (HR = 2.19, 95%CI = 1.6–3.1, *P* < 0.001), and *CDKN2A* (HR = 1.85, 95%CI = 1.2–2.9, *P* = 0.006) were associated with worse OS (Fig. 2-C-D). *KRAS* mutation detection in LB for metastatic disease was associated with worse OS (median 14.5 vs. 31.3 months, HR = 2.7, 95%CI = 1.7–4.3, *P* < 0.001; Fig. 2-E) but the OS difference was not significant in localized disease (Figure S1-A). Notably, in metastatic cases with *KRAS* mutation detected by tumor tissue testing, *KRAS* detection in LB was associated with worse OS (HR = 2.57, 95%CI = 1.42–4.63, *P* = 0.002; Fig. 2-F). The most frequent *KRAS* mutation detected was *KRAS*^*G12D*^ (*N* = 66, 41%), followed by *KRAS*^*G12V*^ (*N* = 58, 36%, Fig. 2-G). *KRAS*^*G12D*^ and *KRAS*^*Q61*^ detection was associated with poorer OS in patients with positive liquid biopsy (Fig. 2-H), which is consistent with our previous findings in patients who had tissues testing [9].

Among 41 patients who underwent multiple LB tests, 25% were initially ctDNA-negative then subsequently tested positive. None of the 22 patients with positive results converted to negative in the subsequent tests. Among 35 patients who received systemic treatment, patients with increased number of detected mutations (*n* = 16) had a trend of worse OS (median OS 22.9 months vs. 26.4 months) compared with decreased number of mutations (*n* = 3; HR = 2.1, 95%CI = 0.48–15.02, *P* = 0.37, Figure S1-B). Patients with increased VAF for *KRAS* (*n* = 18, median OS = 18.7 months) or *TP53* (*n* = 13, median OS = 22.9 months) showed a tendency towards worse OS compared to patients with decreased VAF of *KRAS* (*n* = 8, median OS = 44.8 months; HR = 2.02, 95%CI = 0.73–5.59, *P* = 0.18, Figure S2-A-C) or decreased VAF of *TP53* (*n* = 4, median OS = 34 months; HR = 1.95, 95%CI = 0.54–7.04, *P* = 0.31, Figure S2-D-F).

## Conclusion

We found that 81.2% (*N* = 186) were LB positive in patients with metastatic disease and 52.4% (*N* = 43) positivity rate in localized disease of PDAC. *KRAS* mutations were detected in 64.6% (*N* = 148) of patients with metastatic disease, while only 16% (*N* = 13) of patients had localized disease (Fig. 1). The detection of any mutation in LB was associated with worse OS in metastatic PDAC (Fig. 2). Moreover, *KRAS* mutations, especially *KRAS*^*G12D*^and *KRAS*^*Q61*^, were associated with worse OS (Fig. 2).

## Electronic supplementary material

Below is the link to the electronic supplementary material.

Supplementary Material 1

## Data Availability

Individual patient-level data are not publicly available to maintain compliance with HIPAA regulations and IRB protocol. Anonymized data are available for non-commercial use from the corresponding author upon request, pending data usage agreement and/or IRB-approved collaboration.
