# Supplementary material for: KRAS mutation detection by liquid biopsy for pancreatic ductal adenocarcinoma
Source: J Hematol Oncol. 2025 Apr 17;18:44. doi: 10.1186/s13045-025-01696-0 (PMC12004821; doi:10.1186/s13045-025-01696-0)
Supplement: Supplementary file 1 — Supplementary Material 1 [file 13045_2025_1696_MOESM1_ESM.docx]

**Online only supplements**

***KRAS* Mutation Detection by Liquid Biopsy for Pancreatic Ductal Adenocarcinoma**

Mahmoud Yousef^1^, Abdelrahman Yousef^1^, Mark W. Hurd^2^, Ashwathy Pillai^3^, Saikat Chowdhury^1^, Rebecca Snyder^4^, Mark Knafl^5^, Ryan L. Lewis^6^, Paul M. Roy^6^, Mohammad Fanaeian^1^, Sali Albarouki^7^, Luca F. Castelnovo^1^, Jennifer Peterson^1^, Brandon G. Smaglo^1^, Robert A. Wolff^1^, Shubham Pant^1^, Jason Willis^1^, Ryan Huey^1^, Michael Overman^1^, Ching-Wei Tzeng^4^, Michael P. Kim^4^, Naruhiko Ikoma^4^, Jess E. Maxwell^4^, Matthew H.G. Katz^4^, Huamin Wang^8^, Anirban Maitra^2,8^, Eugene Koay^9^, Ethan B. Ludmir^9,10^, Anthony Chen^1^, Camila Lopez^1^, Haoqiang Ying^11^, John Paul Shen^1^, Dan Zhao^1^

**Affiliations**:

1. Department of Gastrointestinal Medical Oncology, The University of Texas MD Anderson Cancer Center, Houston, TX, USA
2. Sheikh Ahmed Center for Pancreatic Cancer Research, The University of Texas MD Anderson Cancer Center, Houston, TX, USA
3. Department of Hospital Medicine, The University of Texas MD Anderson Cancer Center, Houston, TX, USA
4. Department of Surgical Oncology, The University of Texas MD Anderson Cancer Center, Houston, TX, USA
5. Department of Genomic Medicine, The University of Texas MD Anderson Cancer Center, Houston, TX, USA
6. Department of Enterprise Data Engineering and Analytics, The University of Texas MD Anderson Cancer Center, Houston, TX, USA
7. Department of Gastroenterology and Hepatology, Baylor College of Medicine, Houston, TX, USA
8. Department of Anatomical Pathology, The University of Texas MD Anderson Cancer Center, Houston, TX, USA
9. Department of Gastrointestinal Radiation Oncology, The University of Texas MD Anderson Cancer Center, Houston, TX, USA
10. Department of Biostatistics, The University of Texas MD Anderson Cancer Center, Houston, TX, USA
11. Department of Molecular and Cellular Oncology, Division of Basic Science Research, The University of Texas MD Anderson Cancer Center, Houston, TX

**Corresponding Author**:

Dan Zhao, MD, PhD

Department of Gastrointestinal Medical Oncology, MD Anderson Cancer Center

1515 Holcombe BLVD, unit 0426, Houston, TX 77030

Phone: 832-750-5964; Email: [dzhao3@mdanderson.org](mailto:dzhao3@mdanderson.org)

**Contents:**

**Supplemental Methods.**

**List of Online only Tables:**

**Table S1**: Baseline Patients characteristics.

**Table S2**: Positive Concordance Rates of Liquid Biopsies and Tissue Testing.

**Table S3:** Number of Patients Who Underwent Tissue Biopsy Testing.

**Table S4**: Genes included in LB panel.

**List of Online only figures:**

**Figure S1**: **OS of patients with KRAS mutations detected by LB in localized** disease and OS association with longitudinal change in number of mutations detected**:** **A**- OS of localized disease patients with positive *KRAS* mutations vs other mutations in LB. **B**- OS with changes in number of mutations detected in LB.

**Figure S2: Longitudinal changes in LB and OS association**: **A**- Number of patients with *KRAS* VAF changes. **B-** Longitudinal change of KRAS VAF in LB. **C-** OS with change in KRAS VAF in LB. **D**- Number of patients with *TP53* VAF changes **E-** Longitudinal change of TP53 VAF in LB. **F-** OS with change of TP53 VAF in LB.

**Supplemental Methods:**

**Study population:**

Patients with histologically confirmed PDAC who underwent in-house non-tumor informed LB testing at MD Anderson Cancer Center from January 1, 2018, to March 1, 2023, were included in the follow-up until March 2024. The Foundry platform (Palantir Technologies, Denver, CO) was used to query the electronic health records (EHR) database[1-3]. This study was approved by the MD Anderson Institutional Review Board (IRB), protocol number 2023-0091. A waiver of informed consent was granted per the USA federal regulation 45 CFR 46.116(f) for this retrospective study. Patient demographics, OS, and clinical and molecular data were extracted from Foundry incorporated tumor registry data[2-4]. Data collection was completed through manual EHR chart reviews. OS was defined based on the initial diagnosis. Tumor staging information was collected at the time of initial diagnosis and at the time of LB. The overall cohort was used to investigate the positivity rate of LB by segregating patients with localized and metastatic disease at the time of LB. Usually, LB was done at first visit and at progression of disease with a median time difference between first contact and liquid biopsy collection of 13 days. A subset of patients who underwent both tumor tissue NGS and LB (n=116) was analyzed for concordance (**Table S3)**. A subset of patients who underwent serial liquid biopsies were analyzed for the dynamics of mutation detection and changes in VAFs with clinical outcomes. Repeated LB were usually done on patients after treatment at disease progression. Information on numbers of mutations detected in LB and VAF were collected and compared between repeated tests in the same patients and different groups. Patients with increased number of detected mutations meant if the number of mutations detected by liquid biopsy increased compared to the previous liquid biopsy in the same patient, while decrease in number of detected mutations in the opposite, while stable number meant that the number of mutations detected did not change compared to the previous liquid biopsy. Increase in VAF is any increase in the VAF of KRAS or TP53 compared to their VAF in the previous liquid biopsy for the same patient

**Molecular testing:**

Molecular testing was performed at MD Anderson’s molecular diagnostics laboratory (MDL), which is College of American Pathologists (CAP) accredited and Clinical Laboratory Improvement Amendments (CLIA) certified. LB is an NGS-based analysis of circulating cell-free DNA (cfDNA) for the detection of somatic mutations in 70 genes and includes copy number changes in 19 genes and gene fusions in 6 genes (**Table S4**). The genomic reference sequence was GRCh37/hg19. The software used for this analysis was Illumina NextSeq Control Software 2.1.0, Illumina Real-time Analysis Software 2.4.11, and Bioinformatics Pipeline 3.3.1. The analytical sensitivity for single nucleotide variations, insertions/deletions, and gene fusions was 0.3% VAF for 30ng of cfDNA input and 1% VAF for 5ng of cfDNA input, considering the depth of coverage at a given base (≥250 reads). For patients who only underwent liquid biopsy testing, since hematopoietic cells can also contribute to cfDNA, the possibility that any mutation detected may represent clonal hematopoiesis of indeterminate potential (CHIP) or a clonal hematologic disorder cannot be ruled out. For the sub-cohort of patients who underwent tumor tissue NGS, the gene panels evolved during the study period with expanding lists of genes. The analytical sensitivity for single nucleotide variations was set to 5% to 10%, taking into consideration the depth of coverage, tumor percentage, and allelic frequency for the mutation. A five-tier classification system was applied to all genetic alterations, and variants classified as pathogenic or likely pathogenic were included[5]. Germline variants were filtered using a normal tissue or blood samples control for 114 patients of the 116 total patients tested using tissue biopsy, for the other 2 patients both analytic findings such as allelic frequency and publicly available reference databases COSMIC version 64 (Catalog of Somatic Mutations in Cancer, Wellcome Trust Sanger Institute, UK) and dbSNP version 137 (National Institute of Health, US) were used[6, 7]. Somatic variants were further annotated by OncoKB Therapeutic Level of Evidence V2 using the R library Oncokb-annotator (v3.4)[8, 9].

**Statistical methods:**

The baseline characteristics of the study population and frequencies of liquid biopsy-detected mutations are summarized. The significance of the median VAF Difference between patients with localized disease and those with metastatic disease was tested using the Mann-Whitney test. Time-to-event data were estimated using Kaplan-Meier curves, and differences in OS were compared using the log-rank test. Cox proportional hazards regression models were used for univariate analyses; a p-value <0.05 was considered significant in the model. Concordance rates were calculated only for the positive cases (detected mutations in LB) in comparison to the mutations found in the tumor tissue (positive concordance rates). All statistical analyses were performed using GraphPad Prism version 9.0 (GraphPad Software, San Diego, California, USA) and RStudio Version 3.1.0 (RStudio, PBC. Boston, MA). All tests were two-sided, and statistical significance was set at p < 0.05.

**Supplemental Tables:**

| **Table S1: Patient characteristics** | | |
| --- | --- | --- |
| **Gender** | **Number** | **Percentage** |
| Female | 129 | 41.5% |
| Male | 182 | 58.5% |
| **Race** |  |  |
| Asian | 21 | 6.8% |
| Black or African American | 29 | 9.3% |
| Hispanic or Latino | 35 | 11.3% |
| White or Caucasian | 220 | 70.7% |
| Other | 6 | 1.9% |
| **Vital status** |  |  |
| Deceased | 199 | 64.0% |
| Alive | 112 | 36.0% |
| **Age at diagnosis (Years)** |  |  |
| Mean (SD) | 64.4 | 9.5 |
| Median (Range) | 64.9 | 37.4-85 |
| **Follow up (Months)** |  |  |
| Median (95%CI) | 34.9 | 30.0 - 47.0 |
| **Overall survival (Months)** |  |  |
| Median (95%CI) | 22.5 | 19.2 - 25.8 |
| **Stage at diagnosis** |  |  |
| I | 38 | 12.2% |
| II | 32 | 10.3% |
| III | 60 | 19.3% |
| IV | 181 | 58.2% |
| **Stage at liquid biopsy** |  |  |
| I | 11 | 3.5% |
| II | 13 | 4.2% |
| III | 58 | 18.6% |
| IV | 229 | 73.6% |
| **Histological grade** |  |  |
| Well differentiated | 10 | 3.2% |
| Moderately differentiated | 68 | 21.8% |
| Poorly differentiated | 67 | 21.6% |
| Unknown | 166 | 53.4% |

| **Table S2: Positive Concordance Rates Between Liquid Biopsies and Tissue Testing*** | | |
| --- | --- | --- |
|  | **Metastatic disease** | **Localized disease** |
| ***KRAS*** | 63% (n=50/80) | 7% (n=2/27) |
| ***TP53*** | 68% (n=43/63) | 33% (n=7/21) |
| ***SMAD4*** | 26% (n=5/19) | 0% (n=0/5) |
| ***CDKN2A*** | 80% (n=8/10) | 0% (n=0/7) |
| ***ARID1A*** | 50% (n=3/6) | 0% (n=0/3) |
| * Denominators are total numbers of patients tested positive for mutation in tissue testing. Numerators are the total numbers of patients tested positive for mutation in LB. | | |

| **Table S3: Number of Patients Who Underwent Tissue Biopsy Testing** | | |
| --- | --- | --- |
|  | **number of patients tested** | **number of mutations detected in each gene** |
| **Total** | **116** | - |
| **Metastatic** | **85** | - |
| **Localized** | **31** | - |
| *KRAS* | 115 | 107 |
| *TP53* | 102 | 84 |
| *SMAD* | 79 | 24 |
| *CDKN2A* | 71 | 17 |
| *ARID1A* | 62 | 9 |
| *BRCA2* | 75 | 7 |
| *ATM* | 78 | 3 |
| *EGFR* | 75 | 2 |
| *BRCA1* | 76 | 2 |
| *GNAS* | 66 | 2 |
| *NF1* | 60 | 2 |
| *PIK3CA* | 66 | 1 |
| *MET* | 63 | 1 |
| *STK11* | 62 | 1 |

| **Table S4: Genes included in the liquid biopsy panel** | |
| --- | --- |
| **Gene** | **Exons (codons) tested** |
| AKT1 (NM_005163) | 3 (16-59), 6 (146-189) |
| ALK (NM_004304) | 18-29 (972-1621) |
| APC (NM_000038) | 2-16 (1-2844) |
| AR (NM_000044) | 1-8 (1-921) |
| ARAF (NM_001654) | 7 (186-233), 14 (474-517) |
| ARID1A (NM_006015) | 1-3 (126-601), 6-8 (721-911), 11-20 (997-2286) |
| ATM (NM_000051) | 8 (301-355), 55 (2671-2717), 63 (2996-3057) |
| BRAF (NM_004333) | 1-18 (1-767) |
| BRCA1 (NM_007294) | 2-23 (1-1864) |
| BRCA2 (NM_000059) | 2-11 (1-2281), 13-27 (2313-3419) |
| CCND1 (NM_053056) | 1-2 (1-138), 4-5 (192-296) |
| CCND2 (NM_001759) | 1-2 (1-137), 5 (241-290) |
| CCNE1 (NM_001238) | 4-6 (38-154), 8-12 (204-411) |
| CDK4 (NM_000075) | 2-8 (1-304) |
| CDK6 (NM_001259) | 2-8 (1-327) |
| CDKN2A (NM_000077) | 1-2 (1-153) |
| CTNNB1 (NM_001904) | 3 (5-81) |
| DDR2 (NM_006182) | 14-17 (577-811) |
| EGFR (NM_005228) | 1-28 (1-1211) |
| ERBB2 (NM_004448) | 1-27 (1-1256) |
| ESR1 (NM_000125) | 5 (366-412), 6-8 (416-596) |
| EZH2 (NM_004456) | 16 (618-649) |
| FBXW7 (NM_033632) | 9-10 (413-548), 12 (619-708) |
| FGFR1 (NM_015850) | 2-4 (1-148), 6-8 (206-359), 10-13 (427-616), 15 (658-681), 17-18 (727-821) |
| FGFR2 (NM_000141) | 2-13 (1-621), 15-18 (663-822) |
| FGFR3 (NM_000142) | 2-18 (1-807) |
| GNA11 (NM_002067) | 5 (202-245) |
| GNAQ (NM_002072) | 5 (202-245) |
| GNAS (NM_000516) | 8-9 (196-240) |
| HNF1A (NM_000545) | 3-4 (176-319) |
| HRAS (NM_005343) | 2-5 (1-190) |
| IDH1 (NM_005896) | 4 (41-138) |
| IDH2 (NM_002168) | 4 (125-178) |
| JAK2 (NM_004972) | 14 (593-622) |
| JAK3 (NM_000215) | 13 (568-596) |
| KIT (NM_000222) | 1-21 (1-977) |
| KRAS (NM_004985) | 2-5 (1-189) |
| MAP2K1 (NM_002755) | 2-3 (27-146) |
| MAP2K2 (NM_030662) | 2-3 (31-150) |
| MAPK1 (NM_002745) | 1-8 (1-361) |
| MAPK3 (NM_002746) | 1-8 (1-380) |
| MET (NM_001127500) | 2-9 (1-755), 10-21 (772-1409) |
| MLH1 (NM_000249) | 12 (347-470) |
| MPL (NM_005373) | 10 (490-522) |
| MTOR (NM_004958) | 2-30 (1-1490) |
| MYC (NM_002467) | 1-3 (1-455) |
| NF1 (NM_001042492) | 2-6 (21-218), 8-14 (244-547), 16-18 (574-751), 20-21 (776-950), 24-28 (1038-1290), 30 (1325-1370), 32-50 (1392-2486), 52-55 (2539-2705), 57-58 (2721-2840) |
| NFE2L2 (NM_006164) | 2 (16-104) |
| NOTCH1 (NM_017617) | 4 (135-248), 8 (419-481, 26 (1529-1673), 34 (2061-2556) |
| NOTCH2 (NM_024408) | 1 (1-25), 34 (2400) |
| NPM1 (NM_002520) | 11 (283-295) |
| NRAS (NM_002524) | 2-5 (1-190) |
| NTRK1 (NM_002529) | 8-10 (284-417), 12 (452-501), 14-15 (545-682) |
| NTRK3 (NM_002530) | 16-17 (573-711) |
| PDGFRA (NM_006206) | 3-7 (17-374), 9-12 (413-596), 14-15 (631-719), 18-20 (814-925), 22-23 (961-1090) |
| PIK3CA (NM_006218) | 2-21 (1-1069) |
| PTEN (NM_000314) | 1-2 (1-55), 4-9 (70-404) |
| PTPN11 (NM_002834) | 3 (46-111), 13 (483-533) |
| RAD51 (NM_002875) | 2-3 (1-75), 5-10 (115-340) |
| RAF1 (NM_002880) | 2-3 (1-107), 5 (142-194), 7-8 (227-288), 10 (331-370), 15-17 (513-649) |
| RB1 (NM_000321) | 1-4 (1-167), 7-8 (203-287), 10-14 (314-463), 16-23 (474-830), 25-27 (841-929) |
| RET (NM_020975) | 9-12 (568-727), 14-16 (798-934) |
| ROS1 (NM_002944) | 31-32 (1694-1789), 34-36 (1853-1980), 38 (2002-2045) |
| SMAD4 (NM_005359) | 3 (84-142), 5-6 (152-263), 8-12 (302-553) |
| SMO (NM_005631) | 5 (307-380), 9 (489-551) |
| STK11 (NM_000455) | 1-9 (1-434) |
| TERT (NM_198253) | 1 (1-73), 5 (651-710) |
| TP53 (NM_000546) | 2-11 (1-394) |
| TSC1 (NM_000368) | 15 (480-666), 23 (992-1165) |
| VHL (NM_000551) | 1-3 (63-214) |

**Supplemental Figures:**

**Figure S1:** OS of patients with *KRAS* mutations vs other mutations detected by LB in localized disease and OS association with longitudinal change in number of mutations detected.

**
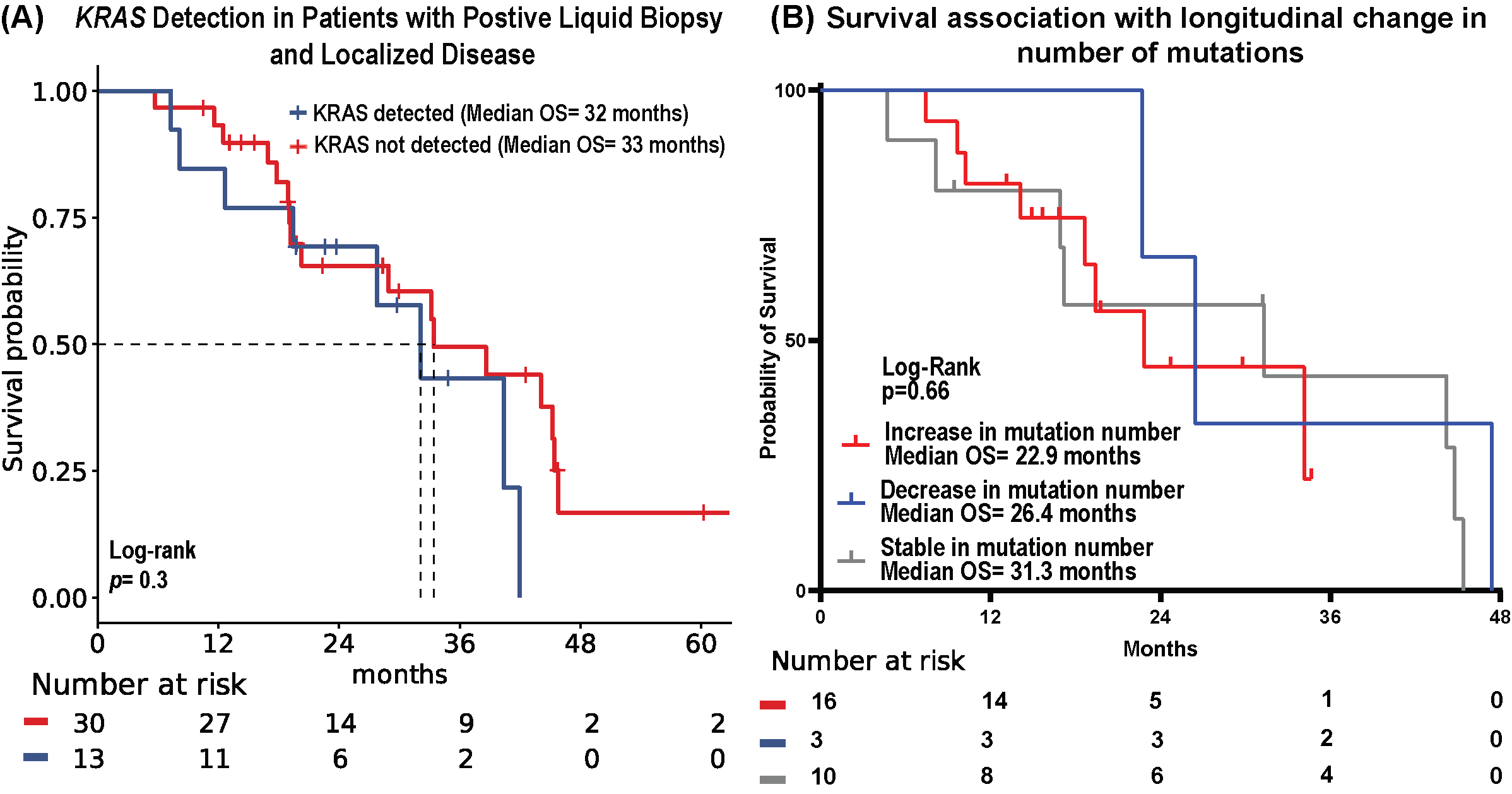
**

**Figure S2: Longitudinal changes in LB and OS association.**

**
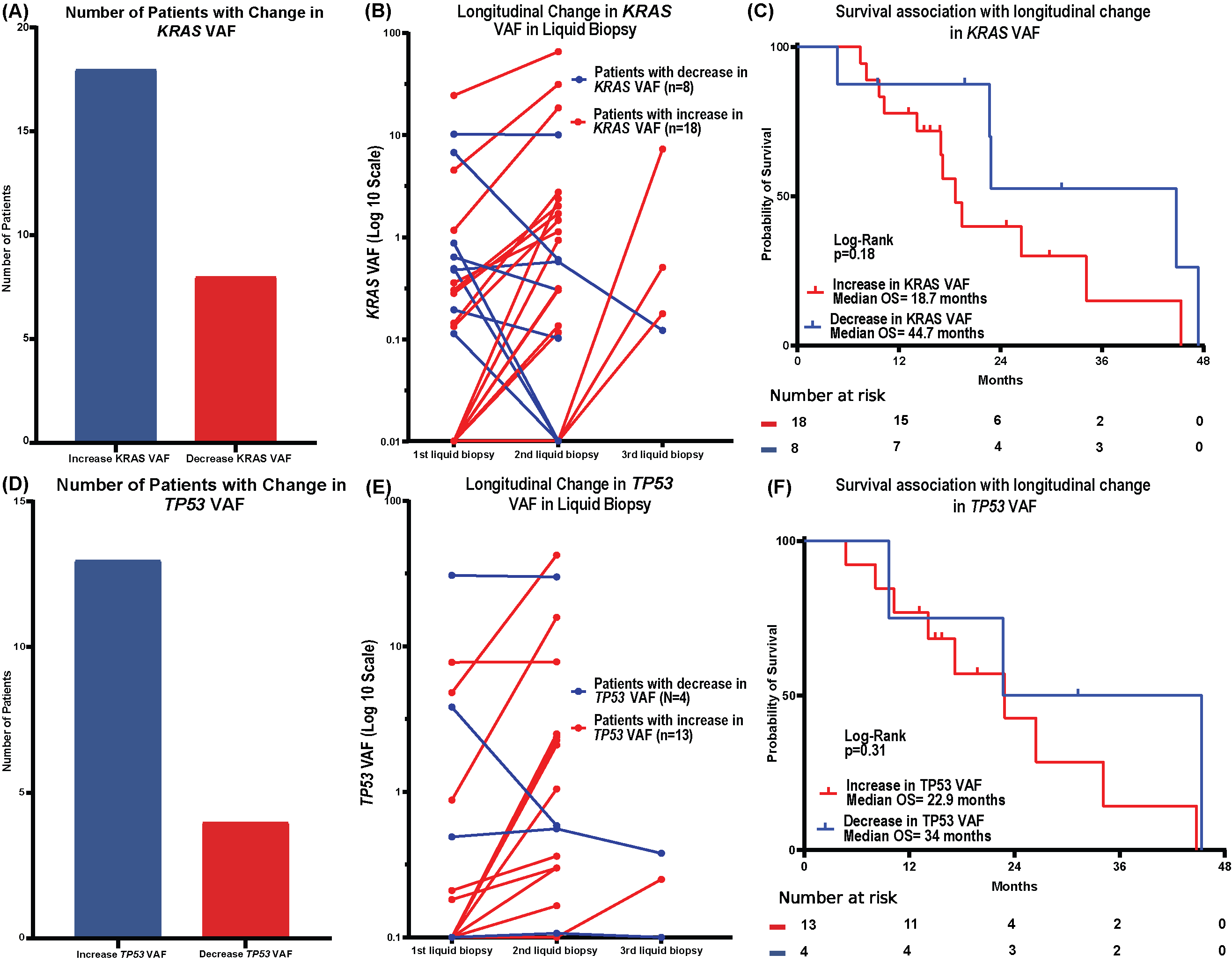
**

**References:**

1. Alfaro-Munoz K, Hallatt G, Sookprasong J, Groot JFD, Lari B, Yu E, Jin J, Futreal A, Ravi V: **Building a data foundation: How MD Anderson and Palantir are partnering to accelerate research and improve patient care**. *Journal of Clinical Oncology* 2019, **37**(15_suppl):e18077-e18077.

2. Goldstein JB, Beird H, Zhang J, Belmont C, Lari B, Mynhier M, Pathak D, Basu P, Jin J, Barbosa G *et al*: **Tackling "big data" for accelerating cancer research**. *Journal of Clinical Oncology* 2016, **34**(15_suppl):e23160-e23160.

3. Bhutiani N, Yousef MMG, Yousef A, Zeineddine M, Knafl M, Ratliff O, Fernando UP, Turin A, Zeineddine FA, Jin J *et al*: **Automated, High-Throughput Platform to Generate a High-Reliability, Comprehensive Rectal Cancer Database**. *JCO Clinical Cancer Informatics* 2024(8):e2300219.

4. Yousef A, Yousef M, Chowdhury S, Abdilleh K, Knafl M, Edelkamp P, Alfaro-Munoz K, Chacko R, Peterson J, Smaglo BG *et al*: **Impact of KRAS mutations and co-mutations on clinical outcomes in pancreatic ductal adenocarcinoma**. *npj Precision Oncology* 2024, **8**(1):27.

5. Richards S, Aziz N, Bale S, Bick D, Das S, Gastier-Foster J, Grody WW, Hegde M, Lyon E, Spector E *et al*: **Standards and guidelines for the interpretation of sequence variants: a joint consensus recommendation of the American College of Medical Genetics and Genomics and the Association for Molecular Pathology**. *Genet Med* 2015, **17**(5):405-424.

6. Sondka Z, Bamford S, Cole CG, Ward SA, Dunham I, Forbes SA: **The COSMIC Cancer Gene Census: describing genetic dysfunction across all human cancers**. *Nature Reviews Cancer* 2018, **18**(11):696-705.

7. Smigielski EM, Sirotkin K, Ward M, Sherry ST: **dbSNP: a database of single nucleotide polymorphisms**. *Nucleic Acids Res* 2000, **28**(1):352-355.

8. Suehnholz SP, Nissan MH, Zhang H, Kundra R, Nandakumar S, Lu C, Carrero S, Dhaneshwar A, Fernandez N, Xu BW *et al*: **Quantifying the Expanding Landscape of Clinical Actionability for Patients with Cancer**. *Cancer Discov* 2024, **14**(1):49-65.

9. Chakravarty D, Gao J, Phillips SM, Kundra R, Zhang H, Wang J, Rudolph JE, Yaeger R, Soumerai T, Nissan MH *et al*: **OncoKB: A Precision Oncology Knowledge Base**. *JCO Precis Oncol* 2017, **2017**.
